# Management of a Facilitated Aesthetic Orthodontic Treatment with Clear Aligners and Minimally Invasive Corticotomy

**DOI:** 10.3390/dj8010019

**Published:** 2020-02-15

**Authors:** Silvia Caruso, Atanaz Darvizeh, Stefano Zema, Roberto Gatto, Alessandro Nota

**Affiliations:** 1 MeSVA, University of L’Aquila, 67100 L’Aquila AQ, Italy; silvia.caruso@univaq.it (S.C.); stefanozemaz@libero.it (S.Z.); roberto.gatto@cc.univaq.it (R.G.); 2 IRCCS San Raffaele Hospital, 20132 Milan, Italy; atanazdarvizeh@gmail.com; 3 Dental School, Vita-Salute San Raffaele University and IRCCS San Raffaele, 20132 Milan, Italy

**Keywords:** orthodontic tooth movement, aesthetics, clear aligner appliances, cortical bone injuries, removable orthodontic appliances

## Abstract

Accelerating orthodontic tooth movement has become a topical issue and the corticotomy seems to be the only effective and safe technique reported in the literature. Simultaneously, aesthetic orthodontic treatment with removable clear aligners has become commonly requested. The aim of this paper is to illustrate the management of facilitated aesthetic orthodontic treatment, a combined approach including piezocision corticotomy and clear aligners for orthodontic treatment. Orthodontic planning for traditional clear aligners should be modified to take advantage of the corticotomy technique in order to facilitate the most difficult orthodontic movements needed to achieve treatment completion, where each aligner will be used for four days rather than 15 days for a total time of four months. A corticotomy with a modified minimally invasive flapless piezocision technique should be performed in both jaws at the same time, before the time window of the orthodontic treatment, where the most difficult orthodontic movements are planned. Treatment planning where difficult orthodontic movements, such as anterior open-bite closure and extraction space closure, are easily managed with clear aligners and are presented as examples of facilitated aesthetic orthodontic treatment application. The combination between aesthetic treatment with clear aligners and modified piezocision corticotomy, if carefully planned, seems to represent a synergy that achieves the current goals of orthodontic treatment. The primary objectives of this combination should be facilitating difficult orthodontic movements and reducing treatment duration.

## 1. Introduction

### 1.1. Background

During the last few decades, accelerating orthodontic tooth movement has become a topical issue [1] and there have been many attempts to shorten treatment duration, including surgical procedures as well as skeletal anchorage [2] and orthodontic movement acceleration [3,4,5].

Among the different techniques described in the literature, the corticotomy seems to be the only effective and safe technique for accelerating orthodontic tooth movement according to the scientific literature [6]. A corticotomy is an intentional injury to the cortical bone that is able to accelerate orthodontic tooth movement and dramatically reduce treatment times because it leads to a biological process called regional acceleratory phenomenon (RAP), which is characterized by intensified osteoclastic activity resulting in osteopenia and increased bone modeling. This reduces the resistance of the dense cortical bone to orthodontic tooth movement, achieving more stable results [7,8,9,10,11].

### 1.2. Aim

Initially, this technique presented significant postoperative discomfort related to its aggressive nature due to the elevation of the mucoperiosteal flaps and to the length of the surgical procedure [6,12]. Nevertheless, recently, the piezocision technique was introduced. This procedure entails small incisions, minimal piezoelectric osseous cuts to the buccal cortex only, and bone or soft tissue grafting, performed under local anesthesia through a tunnel approach [13,14] and overcoming most of the disadvantages of traditional corticotomy.

Simultaneously, orthodontic treatment with removable clear aligners has become an increasingly common treatment choice because of the increasing number of adult patients that desire aesthetic and comfortable alternatives to conventional fixed appliances [10,15,16,17], or when there is a temporomandibular joint disease and fixed appliances are not indicated [18,19]. Clear aligners can also have a bite effect on the activity of the masticatory muscles [20].

Today, clear aligner treatment is also requested in children to treat cross-bites or other malocclusions [21,22,23].

Aesthetics and treatment duration are generally the two main expectations for adult patients when requesting orthodontic treatment. The aim of this study is to illustrate the management of a combined approach with piezocision corticotomy and clear aligners for orthodontic treatment.

## 2. Case Reports

### 2.1. Subject Selection

In order to undergo facilitated aesthetic orthodontic treatment (FAOT), including applying a corticotomy surgical technique or orthodontic clear aligners treatment (Invisalign, Align Technology, San Josè, CA, USA), each subject should be carefully evaluated. A cone-beam computed tomography should be added to the standard orthodontic examination (anamnesis, extraoral and intraoral examination and photographs, dental casts, orthopantomography, cephalometry).

In particular, the following indications and contraindications to the surgical–orthodontic approach should be checked.

Indication to FAOT are: healthy periodontium with absence of bleeding on probing, absence of severe dental crowding, the need to perform difficult orthodontic movements with high risk of relapse (i.e., distalization; open bite closure). 

Contraindications to the surgical procedure are: root crowding, thin gingival biotype, active periodontal disease, corticosteroid therapy, systemic or local contraindications to oral surgery.

### 2.2. Orthodontic Planning

Traditional planning for clear aligners should be modified to take advantage of the corticotomy technique in order to facilitate the most difficult orthodontic movements needed to achieve treatment completion. After performing polyvinylsiloxane or digital impressions of the dental arches and sending it to the manufacturer, proper orthodontic planning using digital software (ClinCheck, Align Technology, San Josè, CA, USA) should be performed and the orthodontist should identify the time window in which the most difficult orthodontic movements have to be performed. The surgical procedure should be planned and executed as the starting point of this period in which each aligner will be used for 4 days rather than 14 days for a total time of 4 months.

Careful planning of the ideal attachments needed to correctly use the aligner to achieve these movements is necessary. All subjects gave their informed consent for inclusion before they participated in the study. The study was conducted in accordance with the Declaration of Helsinki, and the protocol was approved by the Ethics Committee of the University of L’Aquila (Document DR206/2013).

### 2.3. Surgical Procedure

**Figure 1:**
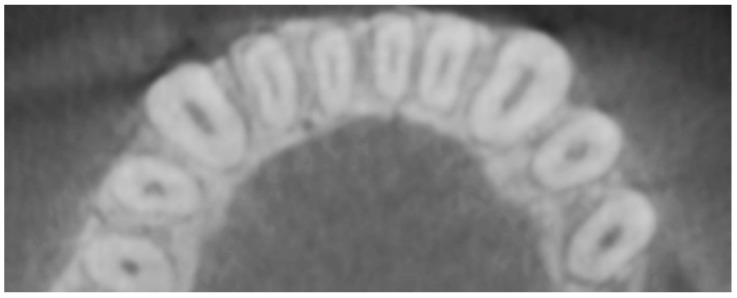
Cone Beam Computed Tomography (CBCT) image of modified piezocision corticotomy between 4-2 and 4-3.

**Figure 2:**
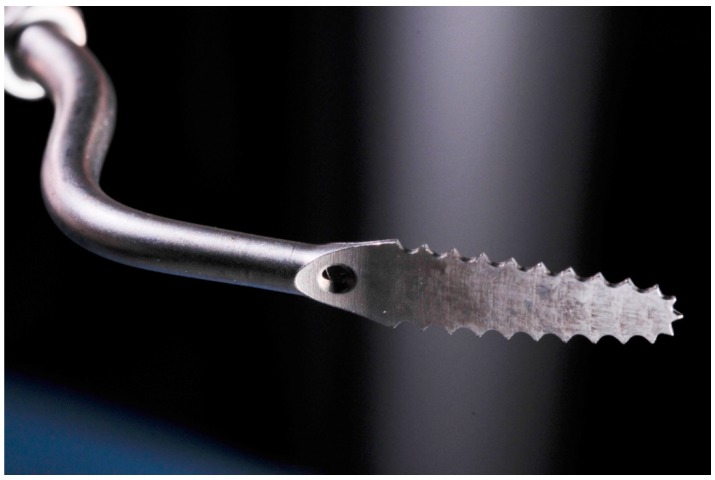
Appropriate sonic insert to perform the modified piezocision technique (thickness: 0.25 mm).

A corticotomy with a modified minimally invasive flapless piezocision technique should be performed in both jaws at the same time before the time window of the orthodontic treatment as defined by the orthodontist. A one-shot antibiotic therapy with 2 g of amoxicillin/clavulanic acid should be administered 1 h before the surgical procedure. A preoperative 0.2% chlorhexidine mouthwash for 1 min should be done [24]. Next, local anesthesia should be administered, delivering articaine 4% with epinephrine 1:100,000 by buccal infiltration. Interproximal corticotomy vertical micro-incisions should be extended through the entire thickness of the cortical layer, just barely penetrating into medullary bone, at a depth of approximately 3 mm for an approximate length of 7–8 mm only on the jaw buccal side (Figure 1). A sonic device (SONICflex, KaVo EWL GmbH, Leutkirch, Germany) is suggested to perform the vertical cuts, using appropriate inserts (SFS 101 and SFS 102, Komet Dental Brasseler, Lemgo, Germany) (Figure 2) with a thickness of 0.25 mm. After the procedure, the areas should be compressed with sterile dressing for 1 min and then sutures with Vicryl 5.0 thread are applied only if bleeding persists.

**Figure 3:**
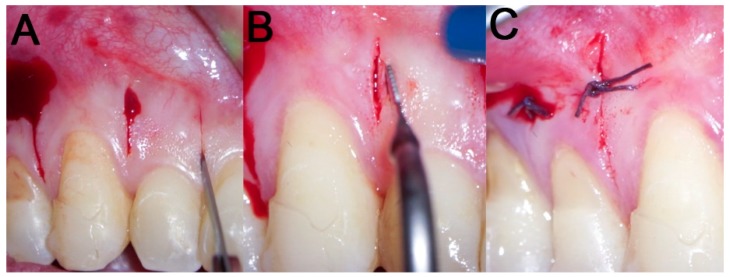
Surgical procedure: (**A**) incision with a 15 blade; (**B**) cortical incision with sonic device and appropriate insert; (**C**) sutures with Vicryl 5.0 thread are applied only if bleeding persists.

The day after surgery, the patient should begin using a chlorhexidine mouthwash 0.2% twice a day for 6 days. No other antibiotic or analgesic therapy is needed. If sutures were needed, they will be removed at the first follow-up visit 7 days after surgery (Figure 3).

#### 2.3.1. Example—Anterior Open-bite

A healthy 20-year-old man came for an orthodontic assessment for an aesthetic improvement of his smile. The extraoral examination revealed satisfactory facial proportions and a good overall aesthetic appearance. All subjects gave their informed consent for inclusion before they participated in the study.

**Figure 4:**
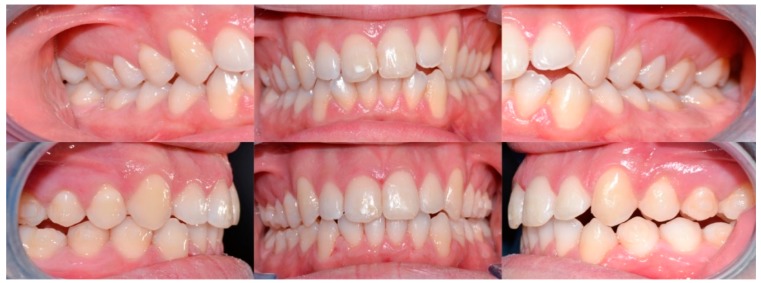
Case 1: initial and final frontal and lateral photo.

**Figure 5:**
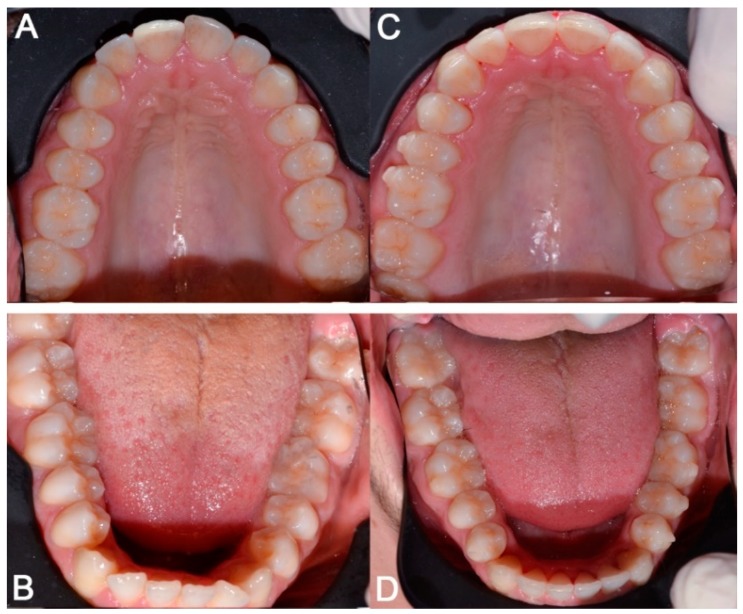
Case 1: initial (**A**,**B**) and final (**C**,**D**) superior and inferior occlusal arches photo.

The intraoral analysis showed a molar and canine Class I relationship on both sides with anterior open-bite on 2-2 (Figure 4 and Figure 5), crowding and light retroinclination of the mandibular incisors; 3-4, 3-5, 4-4 and 4-5 are affected by rotation and increased lingual torque. The patient had good oral hygiene and periodontal health except for the presence of some low gingival recessions. 

**Figure 6:**
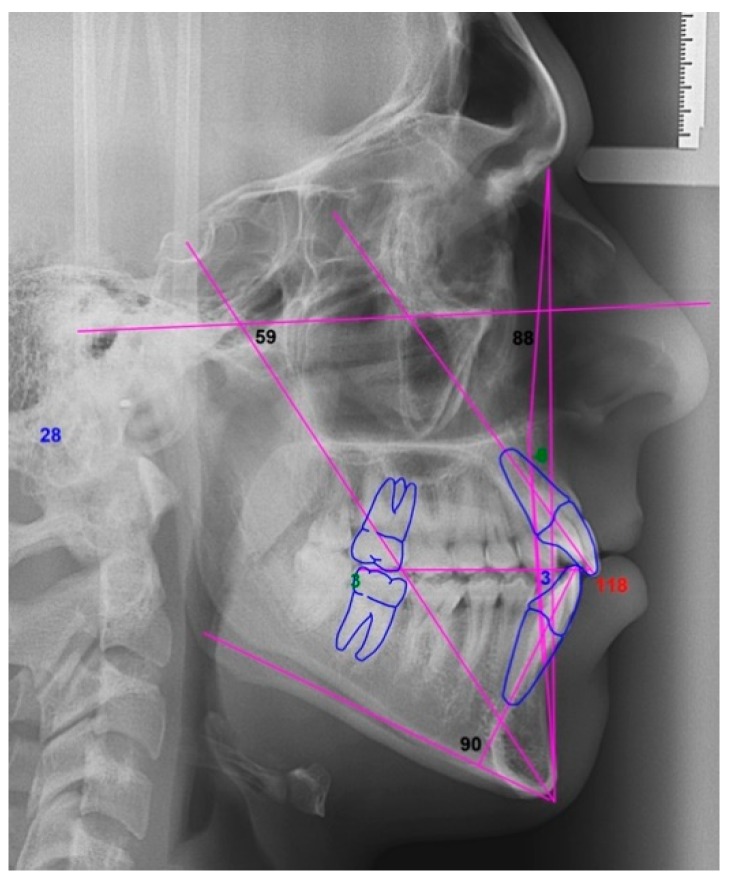
Case 1: cephalometric image.

Orthopantomography and cephalometry (Figure 6) were used for a radiographic evaluation of the case.

The patient’s desire was to have a pleasant smile without applying both conventional or lingual brackets because of their negative impact on aesthetics and comfort. Thus, the treatment objective was to correct the anterior open-bite of 2-2 and to obtain a correct teeth alignment with an aesthetic orthodontic treatment using clear aligners.

**Figure 7:**
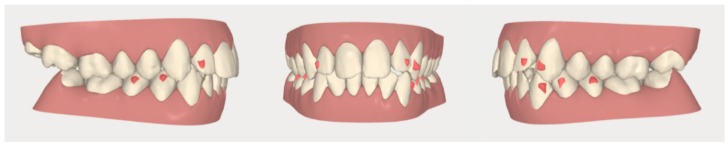
Case 1: initial and final digital frontal and lateral images showing the adopted attachment placement.

As the adult open-bite closing movement of 2-2 is difficult and highly subjected to relapse [25,26], a combined corticotomy and aligners FAOT was planned to achieve this objective and reduce the total orthodontic treatment time. Cone Beam Computed Tomography (CBCT) evaluation and proper aligners were performed as part of the digital orthodontic plan. An optimized attachment was positioned on the 2-2 to obtain the necessary extrusion and torque. In fact, the aligner can apply perpendicular forces on the active surface of these attachments that determine the ideal extrusive movement of the anterior teeth (Figure 7). Optimized attachments were also placed on other elements that needed significant rotations and torque corrections.

**Figure 8:**
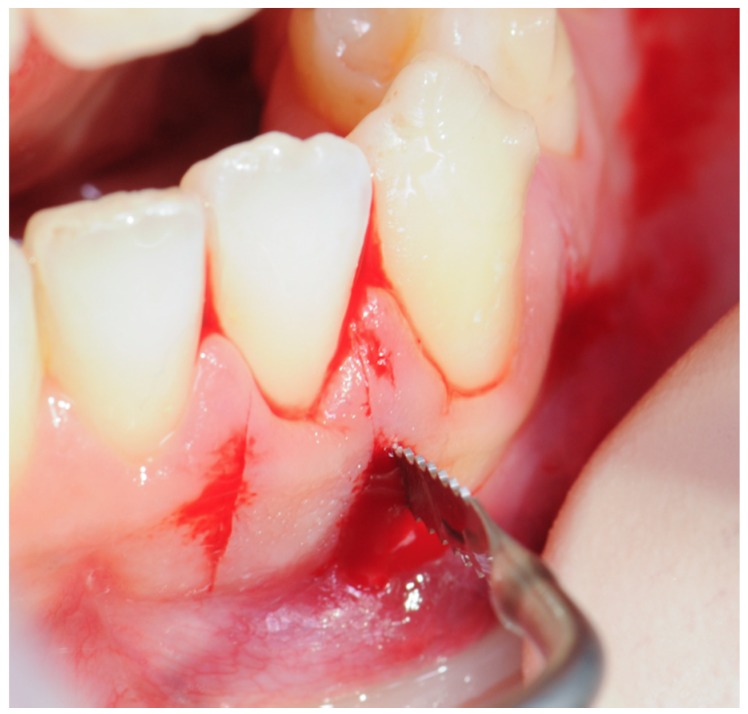
Case 1: modified piezocision corticotomy detail.

**Figure 9:**
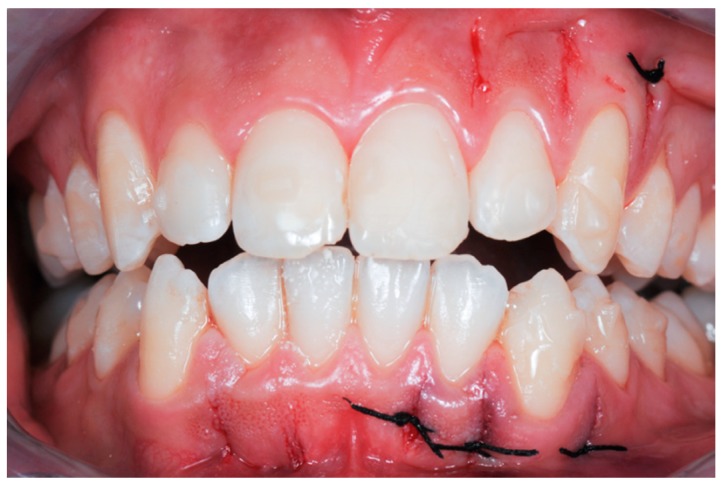
Case 1: photos after the surgical procedure; sutures with Vicryl 5.0 thread were applied only where bleeding persisted.

**Figure 10:**
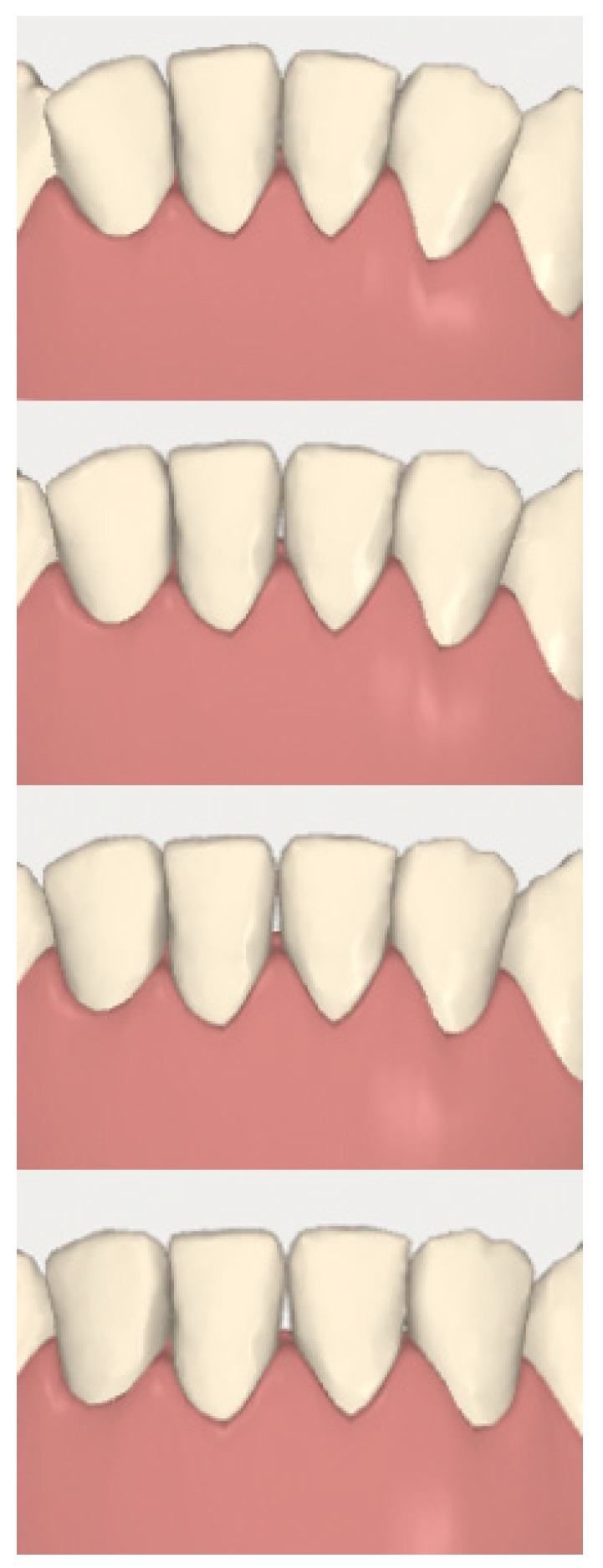
Case 1: digital sequential (up to down) detail of the difficult radicular movement of 3-2 and 4-2.

Before applying the first aligner, the corticotomy was performed distally and mesially to 2-2 and 2-3 to facilitate the open-bite closure and distally and mesially to 3-2, 3-3 and 4-2 that required a difficult radicular movement and rotation in the mandibular arch (Figure 8, Figure 9 and Figure 10). The corticotomy was performed immediately before the first aligner was placed because the difficult movements started with the beginning of the orthodontic treatment and the planned whole orthodontic treatment duration, changing the aligners every 4 days, was of 3 months and 3 weeks instead of 13 months (which would be the required treatment time if changing aligners every 14 days).

#### 2.3.2. Example 2—Extraction Space Closure

A healthy 38-year-old man came in as he was interested in an aesthetic improvement of his smile auspicating to an aesthetic orthodontic treatment option with clear aligners. The extraoral examination revealed satisfactory facial proportions.

**Figure 11:**
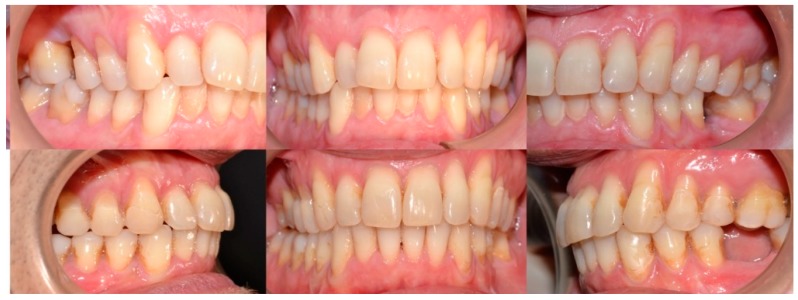
Case 2: initial and final frontal and lateral photo.

**Figure 12:**
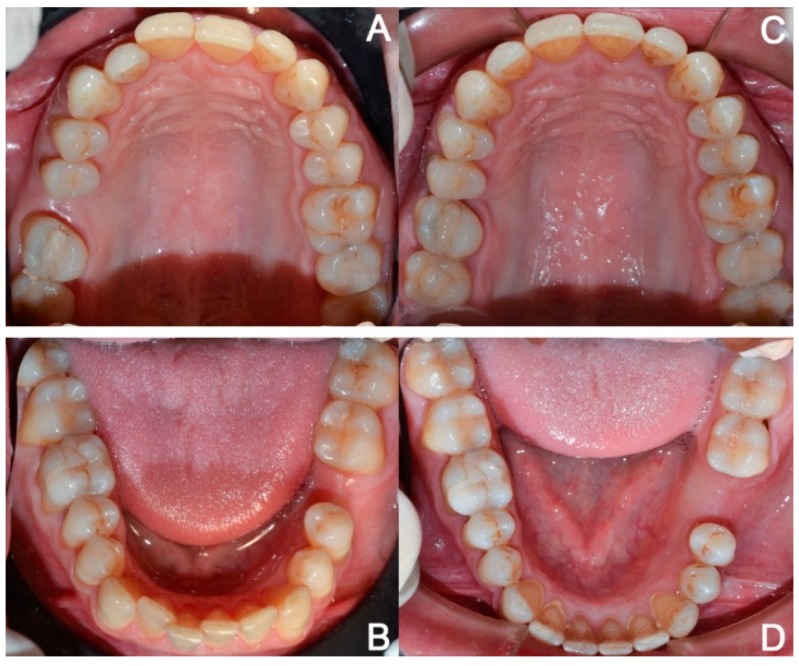
Case 2: initial (**A**,**B**) and final (**C**,**D**) superior and inferior occlusal arches photos.

**Figure 13:**
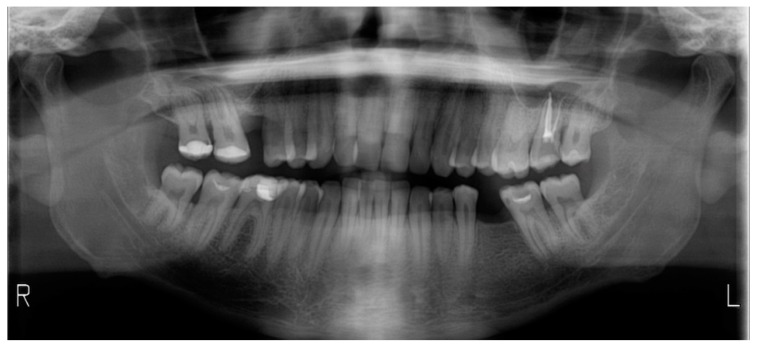
Case 2: orthopantomography.

**Figure 14:**
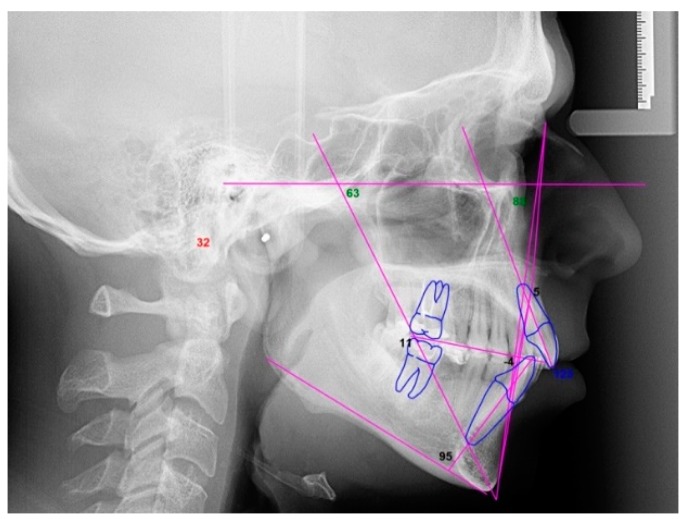
Case 2: cephalometric image.

The intraoral analysis showed a molar (2-6) and canine Class I relationship on both sides, absence of 1-6 and 3-6 and superior and inferior moderate crowding (Figure 11 and Figure 12). The patient had good oral hygiene with absence of bleeding on probing and gingival inflammation but some elements showed moderate gingival recessions. Orthopantomography (Figure 13) and cephalometry (Figure 14) were used for a radiographic evaluation of the case.

**Figure 15:**
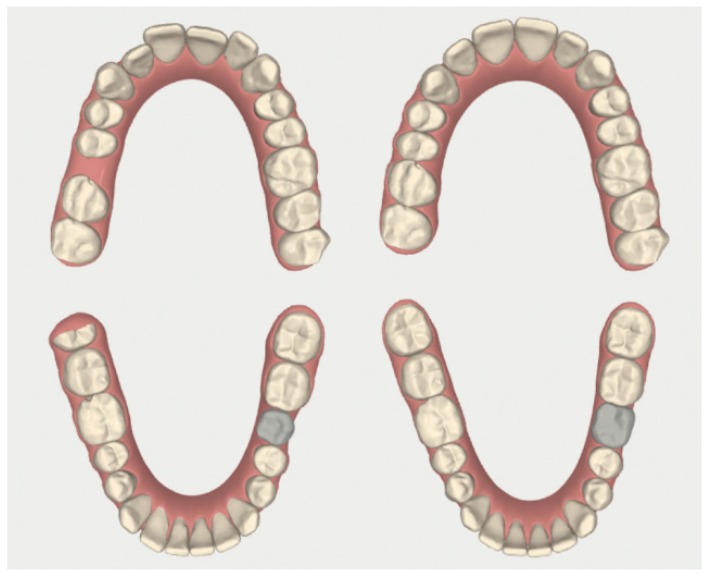
Case 2: initial and final digital occlusal images.

**Figure 16:**
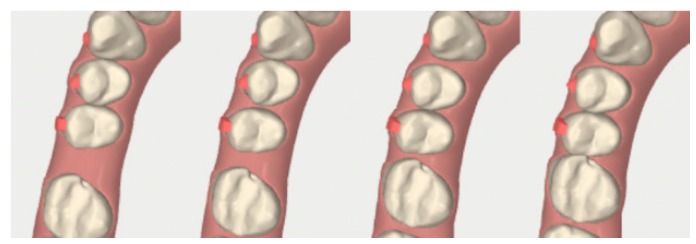
Case 2: digital sequential (left to right) detail of the facilitated space closure orthodontic movement.

**Figure 17:**
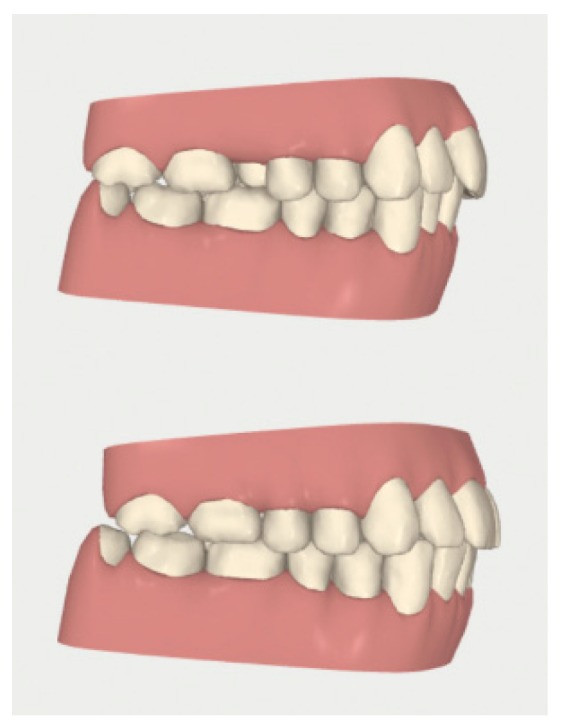
Case 2: digital lateral views before and after the space closure.

After a CBCT evaluation and an accurate analysis on the digital software (Figure 15), the treatment objective was to align the arches by a distalization of the first quadrant, mesialization of 1-7 and 1-8 (Figure 16 and Figure 17) and a proinclination of the inferior incisors. We planned to insert a dental implant at the 3-6 site. 

**Figure 18:**
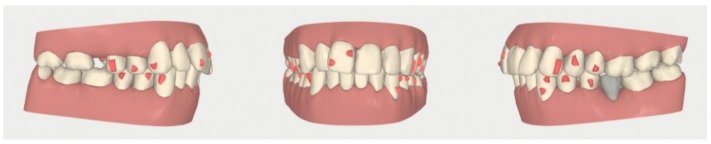
Case 2: initial digital frontal and lateral images showing the appropriate attachment placement for achieving the space closure.

The vertically optimized attachment was positioned on 1-5 to achieve an ideal distalization movement and provide anchorage for the molar mesialization (Figure 18).

**Figure 19:**
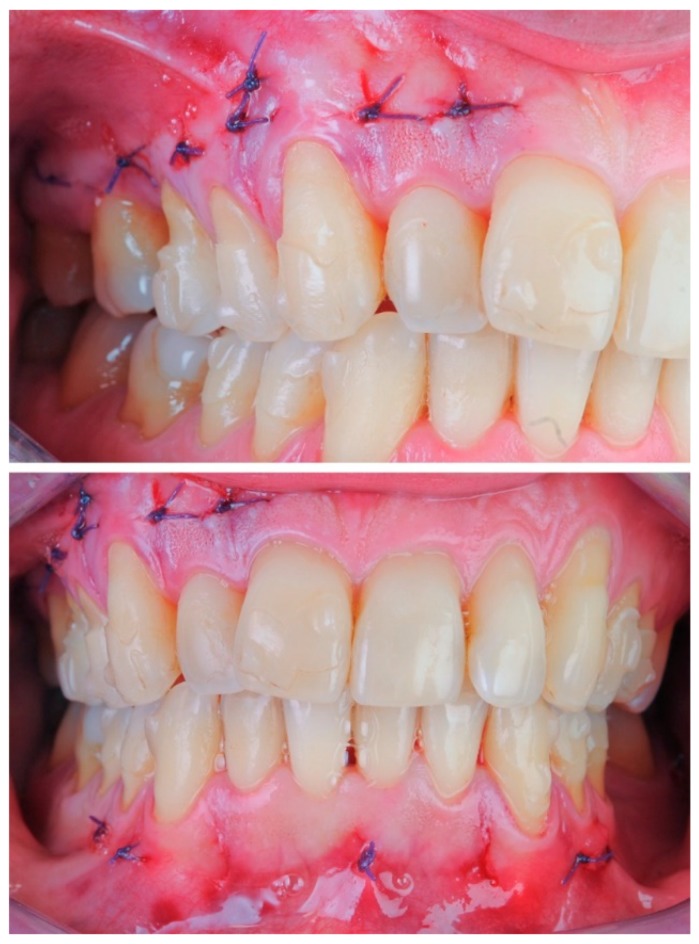
Case 2: photographs after the surgical procedure; sutures with Vicryl 5.0 thread were applied only where bleeding persisted.

Before applying the aligner, number 6 interdental corticotomy incisions were performed in the first quadrant, starting from the distal site of the central incisor to the distal site of the second molar (to facilitate the difficult molar mesialization and premolar distalization movements) and in the mandibular arch starting from the distal site of the lateral incisors to the mesial site of first premolars and between the central incisors (Figure 19). The case was completed in nine months and three weeks instead of 19 months and three weeks (which would have been the required time for changing aligners every 14 days).

## 3. Discussion

Reduced aesthetic impact and treatment duration are the current principal orthodontic expectations for most patients [15,27]. A corticotomy FAOT represents an ideal solution to achieve these goals [1,10].

The regional acceleratory phenomenon (RAP) induced by corticotomy is characterized by a transient increase in bone turnover and a decrease in trabecular bone density that lasts approximately four months [11,28]. During this time window, orthodontic movements are accelerated and achieve more stable results [7,8,9].

Applying this technique to clear aligners therapy allows us to facilitate the most biomechanically complicated movements that must be included in a time window of approximately 30 aligners. In fact, after the surgical procedure, aligners can be changed after four days rather than 14, and this will determine a significant total treatment time reduction of about 10 months because 30 aligners will be changed in that time span instead of eight. It is possible to plan this time window to start with the beginning of the whole treatment or to coincide with the starting point of difficult orthodontic movements as shown in the example cases in this study. Furthermore, the cortical incisions will be performed only in conjunction with the elements that are required to facilitate and accelerate orthodontic movement or to increase the long-term stability of the obtained occlusal changes [7,8,9], apparently increasing the predictability of the effect of the aligners. For this purpose, the accurate choice of the composite attachments and digital workflow planning is fundamental to achieve the planned movements by correctly expressing orthodontic forces on the correct elements [29].

The piezocision technique significantly reduced the disadvantages of traditional corticotomy that presented significant postoperative discomfort [6,14]. By using a sonic device with inserts with a minimal thickness and limiting the incisions to the selected areas, those contraindications are further reduced, making the surgical procedure compatible with patient comfort.

Furthermore, orthodontic treatment with clear aligners guarantees better periodontal health compared to fixed orthodontic appliances [30,31,32,33,34]. Despite this, oral hygiene and periodontal status should be regularly checked after surgery and throughout the period of orthodontic movements [1,27], particularly in patients that smoke [35].

It should be also stated that during the treatment, no adverse effects were reported and no negative effects on the periodontal tissue were observed after the treatment.

Showing only clinical cases is obviously a limitation of the present study; indeed, the successful results obtained are related to patient history and case selection and may not reflect the clinical outcomes for all patients. Clinical studies should be published to analyze the impact of corticotomies in increasing the predictability of orthodontic tooth movement, as the acceleration was already demonstrated [6].

## 4. Conclusions

The combination of aesthetic treatment with clear aligners and modified piezocision corticotomy, if carefully planned, seems to represent a synergy that achieves the current goals of orthodontic treatment with a satisfactory control of moderately difficult orthodontic movements. The primary objective of this combination should be facilitating difficult and less predictable orthodontic movements and reducing the treatment duration; simultaneously, the long term risk of relapse should be reduced. Clinical trials should be conducted for a detailed study of the efficacy and limits of this combined technique.
